# Wandering Spleen, A Rare Cause of Acute Abdomen: A Case Report

**DOI:** 10.1002/ccr3.71204

**Published:** 2025-10-08

**Authors:** John Kanyiri Yambah, Kojo Amanfo Ninson, Abdul Samed Sulemana, Richardson Edwin Annan, Vishnu Nene Limon Abayateye, Gifty Aba Donkor, Moses Dokurugu, Ninnang Tobias

**Affiliations:** ^1^ Department of Surgery, University Hospital University of Education Winneba Ghana; ^2^ Department of Surgery Mampong Government Hospital Mampong Ghana; ^3^ Department of Internal Medicine Korle Bu Teaching Hospital Accra Ghana; ^4^ Department of Surgery Tamale Teaching Hospital Tamale Ghana; ^5^ Department of Internal Medicine Holy Family Hospital, Techiman Ghana

**Keywords:** displaced spleen, ectopic spleen, splenectomy, splenic torsion, wandering spleen

## Abstract

A wandering spleen (WS) is rare and often presents a diagnostic challenge to clinicians. We report a WS in a 16‐year‐old who presented with abdominal pain, vomiting, and peritonism. Imaging (ultrasound and CT scans), which usually facilitates a prompt diagnosis, was inconclusive, and the diagnosis was made by exploratory laparotomy.

## 1 Introduction

Wandering Spleen (WS) is a medical condition identified by the abnormal location of the spleen within the abdominal cavity, distinct from its usual position in the splenic fossa. This rare entity has an incidence of less than 600 descriptions since it was first encountered by Horne in 1667 [1, 2] and accounts for less than 0.5% of all splenectomies. The instances of WS are either congenital or acquired, all arising from the absence or laxity of the supporting ligaments of the spleen [3, 4, 5]. WS may be part of a complex of congenital splenic anomalies such as polysplenia or wandering ectopic spleen, that remain clinically silent until uncovered incidentally or during autopsy [6]. The absence and laxity can make the spleen liable to torsion [4, 7], with a resultant complication of either an infarction, necrosis, or abscess formation. Very few cases of this rare entity have been described among Africans [1, 5, 8, 9] and to the best of our knowledge, only one other case has been described in Ghana [10]. Occasionally, routine physical examination may reveal a mass in the abdomen, which may then be further defined by Ultrasonography and Computed Tomography (CT) scans. WS mimics many abdominal tumors and may be misdiagnosed as Burkitt's lymphoma [11], twisted ovarian cyst [5], tropical malaria splenomegaly, or other abdominal tumors [9]. Splenic torsion, infarction, and microabscess formation, which commonly complicate WS, make it a key differential diagnosis when considering an acute abdomen in the presence of palpable masses. WS has been reported to cause gastric volvulus, gastric outlet obstruction, and pancreatitis [2, 10, 12]. Whether open or laparoscopic, surgery is the mainstay of management, with considerations for either a splenectomy or splenopexy. Splenectomy is the treatment choice when there is infarction and necrosis, and the spleen is deemed to be nonviable. In a few instances, conservative nonsurgical management has been reported as successful [5]. Postsplenectomy, patients are either given benzathine injections as prophylaxis or vaccinated against *Haemophilus influenzae* , *Meningococcus*, or *Pneumococcus* because of the critical role of the spleen in the immune protection against these organisms [2] and further decrease the possibility of overwhelming postsplenectomy infections (OPSI).

We present a case of WS in a 16‐year‐old African Ghanaian female who presented with a two‐week history of abdominal pain diagnosed as an acute abdomen secondary to an ovarian mass with torsion but which turned out to be a WS.

## 2 Case History/Examination

A 16‐year‐old female presented with abdominal pain and vomiting of 2 weeks' duration. Two days before the presentation, she developed a high‐grade fever. She was initially seen a month earlier for similar abdominal pain and was managed as presumed peptic ulcer disease. Her past medical history was significant for a clinical diagnosis of peptic ulcer for the preceding 3 years, and her surgical history was insignificant. Examination revealed a tender right hypochondrial mass with generalized abdominal tenderness and rebound tenderness.

## 3 Methods (Differential Diagnosis, Investigations, and Treatment)

A diagnosis of an acute abdomen was made with a differential diagnosis of peritonitis arising from a twisted ovarian mass.

A complete blood count showed a leukocytosis with a left shift and thrombocytopenia (white blood cell count 12.8 × 10^9^[4–10 × 10^9^], neutrophil count (80%) [40–70], and platelet count 111 × 10^9^ [150–400 × 10^9^]). A blood film for malaria parasites was negative.

An ultrasound confirmed a solid mass in the right lower quadrant but was not conclusive of its origins. A plain noncontrast abdominal CT scan suggested a solid pelvic mass extending into the right flank.

She was started on intravenous antibiotics (Ceftriaxone 2 g daily for 48 h, Gentamicin 80 mg daily for 48 h, and Metronidazole 400 mg 8 hourly for 48 h) and resuscitated for an exploratory laparotomy.

**FIGURE 1:**
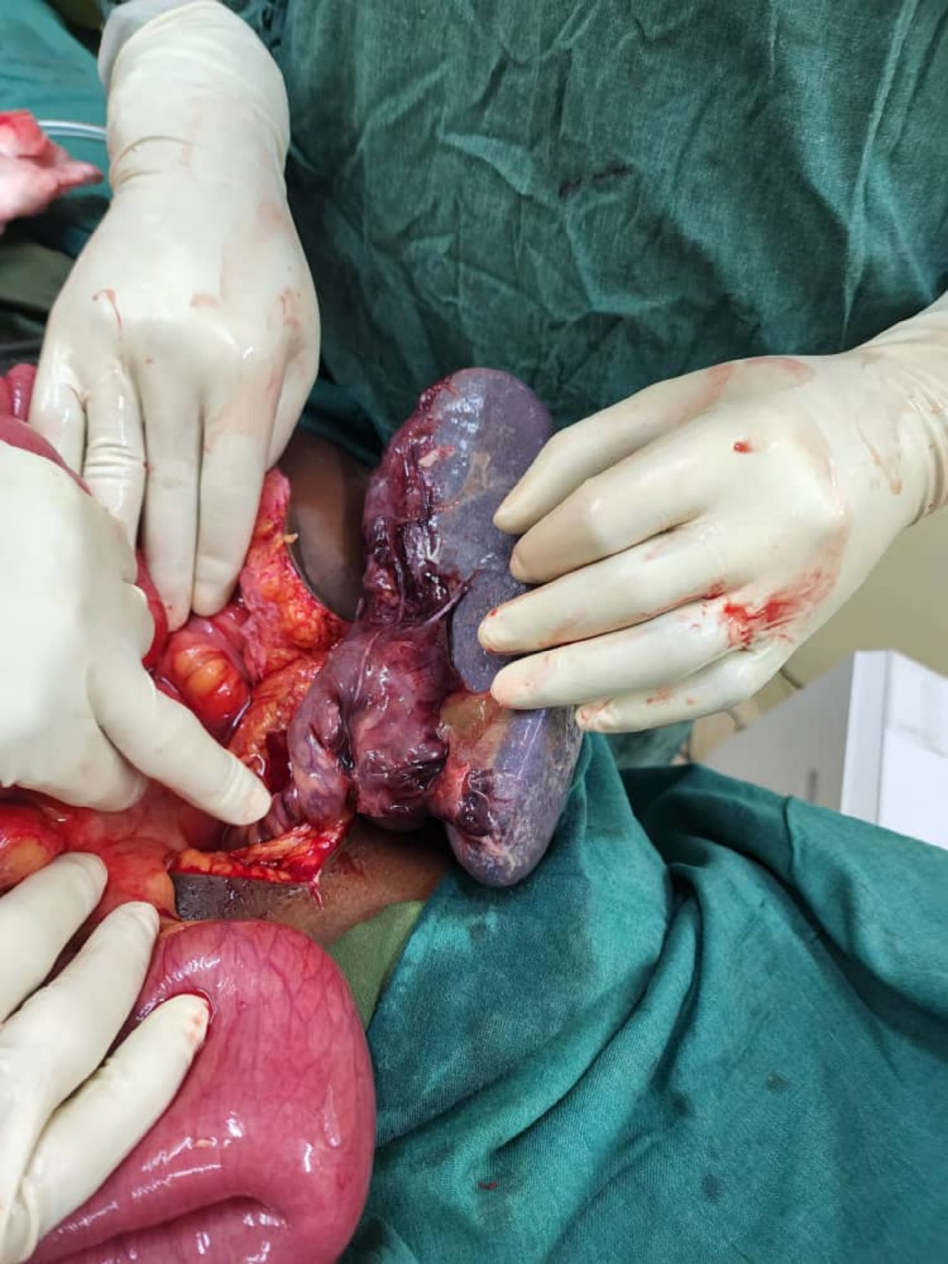
Intraoperative findings of a torsion of the spleen with areas of thrombosis.

**FIGURE 2:**
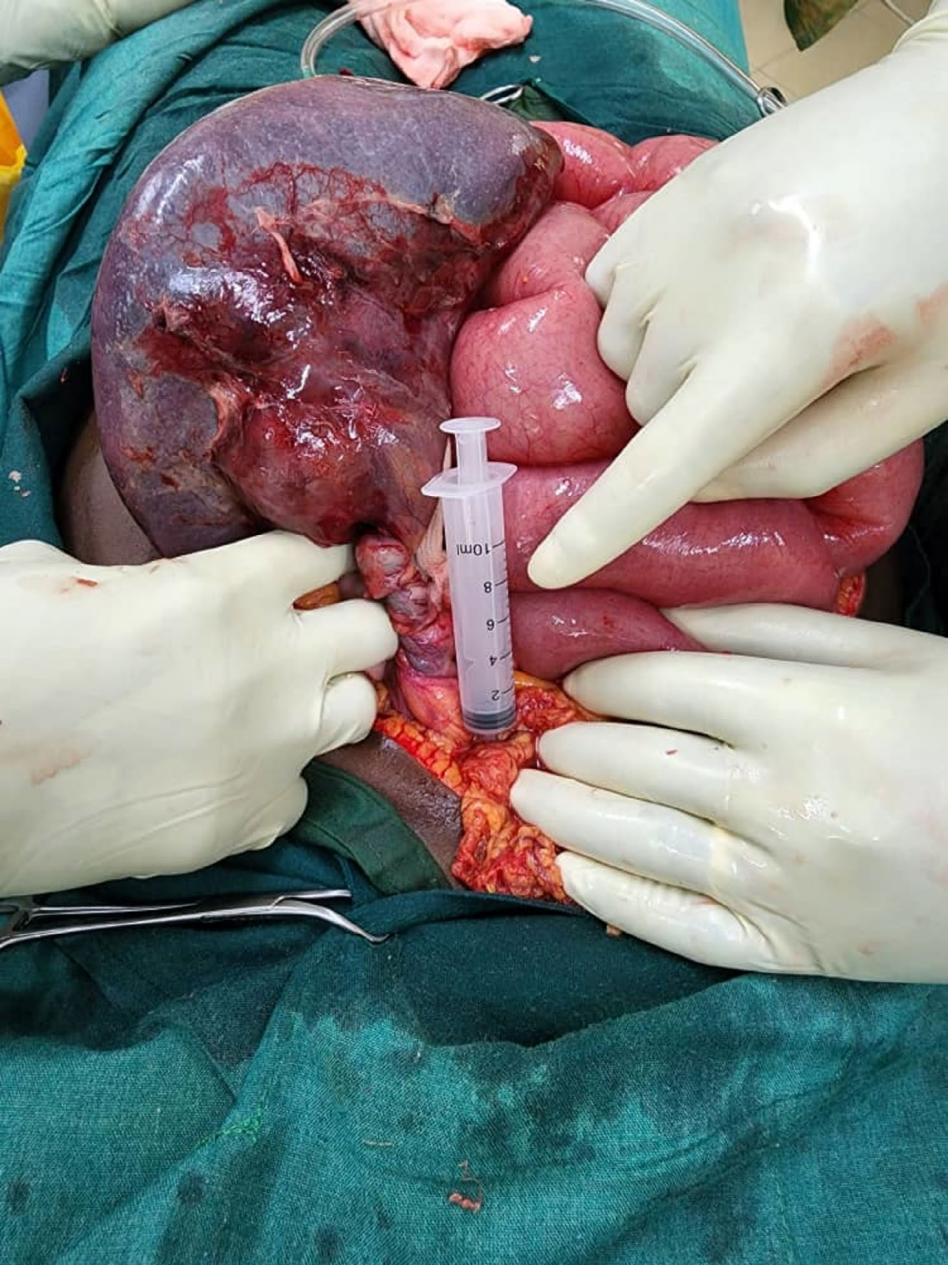
Another view showing the twisted pedicle and part of the tail of the pancreas.

At laparotomy, the findings were an enlarged spleen that measured 12 × 20 × 12 cm and was located in the mesentery along the ascending colon with no lieno‐phrenic and lieno‐colic ligaments. Some portions of the spleen were thrombosed. This is shown in Figure 1. The peritoneum had about 800 mL of sero‐sanguinous fluid. Part of the pancreas was seen at the base of the twisted pedicle, but it was grossly normal, as shown in Figure 2.

A splenectomy was performed from the base of the twisted pedicle to reveal a spleen weighing 0.945 kg. Detorsion was not considered beneficial, and a splenectomy was strongly favored because of the possibility that other splenic pathology could account for the massive splenomegaly.

After surgery, she received maintenance intravenous fluids and antibiotics. The antibiotics comprised intravenous (Ceftriaxone 2 g 12 hourly for 72 h, Gentamycin 80 mg daily for 72 h, and Metronidazole 500 mg 8 hourly for 72 h) together with analgesics, suppository Diclofenac 50 mg 8 hourly for 5 days, and intravenous Paracetamol 500 mg 8 hourly for 48 h. Her antibiotics were switched to oral antibiotics after post‐op day 4, and she was discharged home on oral Amoxicillin + Clavulanic acid 625 mg twice daily for 7 days and oral Metronidazole 400 mg three times daily for 7 days.

The histopathology of the excised spleen revealed infarction with microabscess formation.

## 4 Conclusions and Outcomes

She made an uneventful recovery, and she was discharged home on post‐op day 5. A booster pentavalent immunization against *Meningococcus*, *Pneumococcus*, and *Haemophilus influenza* was given 8 weeks after discharge.

There were no complications after 6 months of follow‐up.

## 5 Discussion

Cases of WS may be difficult to establish because the fundamental elements of a diagnosis, a spleen elsewhere except the splenic fossa, are hinged on operator‐dependent competence and a very high index of suspicion. This is more so in resource‐poor settings where skilled radiology services are often unavailable to the primary care physician. This is compounded by a myriad of other abdominal tumors that may also appear similar to WS.

This condition, which may be congenital or acquired, has a bimodal distribution with a female‐to‐male ratio of 7 to 1 [2]. The initial peak occurs between birth and 10 years and then again between the ages of 20–40 years [2]. However, the condition may present from as early as 4 months of age to as late as 87 years.

The aetiogenesis arises from the absence, weakness, laxity, or lack of all or some of the supporting ligaments responsible for affixing the spleen in the splenic fossa [5].

Consistent with many of the cases described [5, 10, 12], ours was found in a 16‐year‐old female. She presented with abdominal pain and vomiting, most likely due to the recent torsion, evidenced by the sero‐sanguinous peritoneal fluid and thrombosed splenic vein. It is also probable that the earlier presumed managements for peptic ulcer disease when she presented with epigastric pain were related to repeated bouts of torsion without necrosis.

Peritonism limited a detailed physical examination of the mass, unlike in other instances where the spleen was well described during physical examination and even in one example where it was ballotable and was observed to change position on lateral shifts [2, 13]. In the index case, because WS is rarely encountered, the clinical diagnosis, as well as the ultrasound imaging evaluation, did not entertain its possibility, even though the whirl sign could easily have led to a diagnosis of WS [14]. An abdominal CT scan reported a well‐defined mass arising from the right pelvis. Peritonism coupled with a significant leukocytosis with differential neutrophilia and a tender mass suggested a twisted pelvic mass probably arising from the right ovary because of the location of the mass. This misdiagnosis is not uncommon, especially for a rare condition like WS [5, 9]. Contrary to suggestions of thrombocytosis as a predictor of necrosis, our patient presented with thrombocytopenia even though the spleen was confirmed by histopathology to have undergone infarction and microabscess formation.

A decision was made for an open laparotomy, which is standard for an acute abdomen at our facility. Further, our facility, a typical practice in a low‐resource healthcare facility, lacked the equipment, skill, and personnel for a laparoscopic intervention as used in other case reports. In our instance, we did not encounter a gastric volvulus or gastric perforation [15] reported as complications by other authors. Although part of the pancreas was seen in the twist of the pedicle, it did not appear inflamed. This contrasts with the case where pancreatitis or pancreatic necrosis was reported in other instances [2]. The pedicle was twisted clockwise 1260 degrees. This falls in line with reported cases of torsion that ranged from 180° to 2160° [1, 16] and may have accounted for the repeated bouts of epigastric pain suffered by the patient.

The size and weight of the spleen suggested intrinsic pathology such as Burkitt's lymphoma, or other lymphoid malignancy, tropical malaria splenomegaly, etc., prompting a request for a histopathology on the excised spleen. However, no such pathology was found in our case except for the microabscess formation, infarction, and necrosis.

Postsurgical thrombocytosis did not occur in our case; therefore, we did not require dipyridamole, contrasting with other scenarios where this was crucial [14]. Even though overwhelming postsplenectomy infection, a fatal potential complication, may arise especially in the first 2 years postsplenectomy, follow‐up for 6 months was without any event.

In consonance with the theory of the spleen being important in the fight against encapsulated organisms and to bolster immunity, we elected to give our patient a booster pentavalent vaccine instead of monthly benzathine injections. This is because of suggestions that the benefits of long‐term antibiotics may be unclear and antibiotic efficacy may be reduced as a result of prolonged antibiotic use [17]. Although there were no complications after 6 months of follow‐up, the patient was educated on the possibility of overwhelming postsplenectomy sepsis (OPSI) and the need to seek prompt medical care in instances of ill health [17]. A summary of relevant literature for this case report is attached as (Table 1).

**TABLE 1:** Selected literature summary: Wandering spleen.

Author(s)	Age/Sex	Presentation	Relevant past medical history	Physical examination findings	Laboratory	Imaging findings			Key results/findings	Surgical intervention	Complications
						Ultrasound	CT	MRI			
Shibiru et al. (2024)	39/F	Chronic abdominal pain‐1 year	On antiretroviral therapy‐18 years, Adult polycystic kidney disease. CS 10 years ago	Visible round mass on the right paraumbilical and lower quadrant areas		Hypoechoic, 13 × 8 cm well‐defined right‐sided abdomino‐pelvic mass, absent spleen in the left hypochondrium	Hypoattenuating, well‐circumscribed lesion with no contrast enhancement located in the right abdomino‐pelvic cavity		The spleen was whitish, distended, and grossly infarcted with its long stalk torsed > 360°	Splenectomy	Pentavalent vaccine in the third week
Koliakos et al. (2020)	25/F	Recurring epigastric pain and vomiting pain	Epilepsy		Elevated CRP and serum lipase		Torsion of the splenic pedicle and torsion of the spleen		A torsion of the splenic vascular pedicle was discovered, secondary to the absence of the splenocolic and splenophrenic ligaments	Splenectomy	
Melkamu et al. (2025)	16/F	Abdominal pain and vomiting		Poorly defined, centrally located large mass that measures around 8 cm by 15 cm with tenderness all over the abdomen	WBC was 12 × 10^6^/L (neutrophilia 80%) Hemoglobin 13.2 g/dL, Platelet 304 × 10^6^/L		Abdominal CT with contrast was done and showed a nonenhancing global infarction of the spleen with early signs of splenic infarction		The spleen measured 15 cm × 10 cm with ischemic changes and was volvulated around 270 degrees clockwise		
There was an accessory spleen in the hilar area	Splenectomy										
Kanani and Sheik (2025)	33/M	Autopsy	Burns	Small‐sized spleens measuring 4.5 × 4.5 × 2.8 cm, 4 × 3.5 × 2.3 cm, and 3.5 × 3 × 2 cm Absent right kidney							
Wang et al. (2025)	3/M	Abdominal pain and vomiting		A firm, mobile mass with a smooth surface was noted in the left upper abdomen, with the lower edge at about 10 cm from the lower margin of the costal arch		Mass in the left upper abdominal cavity and absence of spleen in its normal position. Hyperechoic, with a significantly decreased color flow on Doppler	Elongated splenic vascular pedicle		Absence of splenic suspensory ligaments, and a 720°‐twist vascular pedicle involving the tail of the pancreas		
Mamadu et al. (2018)	26/F		G3 P2 at 15 weeks of gestation	Mobile tender mass in the right iliac fossa	The hemoglobin 9 g/dL, and the white blood cell count 12.0 × 10^6^/L and the platelet count was 370 × /L			Right hypochondrial spleen	Ischemic vascular pedicle	Splenectomy	
Salvador et al. (2023)	29/M	5‐year history of progressive abdominal swelling				Epigastric solid mass measuring 20 × 15 cm, highly vascularized with internal calcification		vascularized lobulated mass utterly fused into the greater omentum.		Splenectomy	
Zhou et al. (2023)	24/F	Abdominal pain‐1 week		Mobile mass arising from the pelvis	Normal		Solid mass in the pelvis	The splenic vessels had a whirl appearance at the hilum by gadolinium‐enhanced magnetic resonance imaging (MRI), which was compatible with a twisted splenic pedicle	The spleen was shown to lack its normal ligaments and suspended by a prolonged vascular pedicle, twisted 2160° in a counterclockwise rotation	Splenectomy	
Umeda et al. (2020)	12/M	Abdominal distention and vomiting		Firm mobile mass with a smooth surface and ill‐defined margins was palpable in the umbilical region		X‐ray abdomen, which revealed two large gas shadows in the gastric region that converted into a single shadow after insertion of a nasogastric tube (NGT), suggestive of GV. The left hemidiaphragm was also seen to be slightly elevated as compared with the opposite side signifying eventration	Displaced epigastric spleen and an ascended left kidney		Endoscopic splenopexy		Revealed a swollen, wandering spleen with associated gastric volvulus, gastric perforation
Masroor and Sawari (2021)	27/F	Abdominal pain, fever, nausea, vomiting, and constipation: 3 days, urine retention	Gastritis 2 years prior	The abdomen was mildly distended, palpable, movable mass was found during the physical examination	Leukocytosis	The ultrasound result was not diagnostically significant	Abdominal CT scan with and without contrast was advised, which showed an empty splenic fossa and mildly enlarged ectopic spleen measuring 15.5 cm in length, present in the right lower abdomen extending into the pelvis resting on the bladder fundus		Mildly enlarged, congested and ischemic spleen with 1800 twisted pedicle was found	Splenectomy	
Asafo Adjaye et al. (2019)	14/F	5‐day history of nausea, vomiting, loss of appetite, fever, headache, constipation, and central abdominal pain	One year history of abdominal pain with 4 admissions	The abdomen was flat but with tenderness around her umbilicus. She had no guarding or rebound tenderness	White cell count of 8.1 × 10/L (83.2% granulocytes), hemoglobin was 12.7 g/dL		The stomach had rotated along its long axis with the antrum rotating anterosuperiorly and the fundus rotating posteriorinferiorly. The stomach was grossly distended. The spleen was located anteriorly and the pancreas was rotated left anterolaterally. There was minimal free fluid seen in the pelvis. There was a severe intra‐ and extrahepatic cholestasis			No splenectomy	Ileal intussusception
Broadies et al. (2010)	11/M, 8/F	Abdominal pain and wasting and a one‐month history of abdominal distension, one year history of abdominal distension+ recurrent abdominal pain	Hemotransfused o/a severe anemia(hb < 3.7 g/dL)	His abdomen was moderately distended with a huge tender mass extending from the right upper quadrant to the right lower quadrant, firm tender mass in the right upper quadrant	FNAC; A fine needle aspiration	A large mass 10 × 20 cm			Infarcted spleen	Open splenectomy	
Gashaw et al. (2025)	40/F	Abdominal pain and vomiting		Palpable mass in the periumbilical and right lower quadrant areas with accompanying regional tenderness.		14 × 10 cm mass in the left lower quadrant, showing echodensity similar to splenic parenchyma, with no color Doppler flow and no visible spleen in its usual anatomical location	Spleen measuring approximately 15 cm in size, located in the lower abdomen. The CT also showed twisting of the splenic vessels at the hilum, resembling a “whirlpool” appearance, with heterogeneous density and no postcontrast enhancement. Additionally, part of the pancreatic tail appeared twisted, though without any change in density.		Spleen located in the right lower quadrant, with an elongated vascular pedicle twisted 1080° counterclockwise, involving the tail of the pancreas	Splenectomy	
Somalwar et al. (2011)	32/F	Abdominal pain‐5 days		Pallor and a palpable, freely mobile, nontender, nonballotable mass in the epigastric region, which shifted left or right with lateral positions		Wandering spleen with splenomegaly and an aneurysm of the splenic artery at the hilum	Viable wandering spleen in the epigastric region with mild splenomegaly (12.2 × 7.0 × 11.5 cm) and a splenic artery aneurysm (2.4 × 2.4 cm)			Splenopexy	Aneurysm of the splenic artery at the hilum
Nungho et al. (2024)	49/F	Abdominal pain, constipation and abdominal distention 6 years	Diagnosed with chronic cystitis in presenting scenario	Palpable firm mobile mass in the right lower abdomen	10.2 g/dL, and other blood indices were within the normal range	Lower abdominal solid mass and the absence of the spleen from its normal location with the impression of an ectopic spleen in the pelvis	Absence of spleen in the splenic fossa and its presence in the pelvic cavity, the uterus with a stretched splenic artery and tortuous splenic vein		Normal appearing spleen	Nonsurgical	The uterus with a stretched splenic artery and tortuous splenic vein
Li et al. (2025)	1/F	Fever, vomiting, diarrhea 2 days	Abdominal distension, reduced bowel sounds		White blood cell count of 42.62 × 10^9^/L, red blood cell count of 3.47 × 10^12^/L, platelet count of 945 × 10^9^/L, routine C‐reactive protein level exceeding 200 mg/L, and high‐sensitivity C‐reactive protein level exceeding 5 mg/L	Inhomogeneous, oval, and ill‐defined mass with a capsule and necrotic areas	Abdominal computed tomography (CT) clarified that the mass was well‐circumscribed and regular, with visible exudation		Splenic pedicle twisted by approximately 720 degrees, necrosis and microabscess formation	Splenectomy	Postsurgical secondary thrombocytosis managed with dipyridamole

Clinicians and Sonographers should have a high index of suspicion of the possibility of a wandering spleen in cases where there is an abdominal mass in the absence of the spleen in the left upper quadrant on imaging studies. This, coupled with symptoms of an acute abdomen, makes WS a strong possibility in diagnostic considerations. Routine abdominal examination as part of preschool medical examinations and imaging may aid early recognition before complications arise.

## Author Contributions

**John Kanyiri Yambah:** conceptualization, supervision, writing – original draft, writing – review and editing. **Kojo Amanfo Ninson:** conceptualization, writing – original draft, writing – review and editing. **Abdul Samed Sulemana:** writing – original draft, writing – review and editing. **Richardson Edwin Annan:** conceptualization, writing – original draft, writing – review and editing. **Vishnu Nene Limon Abayateye:** writing – original draft, writing – review and editing. **Gifty Aba Donkor:** conceptualization, writing – original draft, writing – review and editing. **Moses Dokurugu:** writing – original draft, writing – review and editing. **Ninnang Tobias:** writing – original draft, writing – review and editing.

## Consent

The authors obtained a signed informed consent for the publication of this case report.

## Conflicts of Interest

The authors declare no conflicts of interest.

## Data Availability

The data that support the findings of this study are available from the corresponding author upon reasonable request.
